# Obstacle Optimization for Panic Flow - Reducing the Tangential Momentum Increases the Escape Speed

**DOI:** 10.1371/journal.pone.0115463

**Published:** 2014-12-22

**Authors:** Li Jiang, Jingyu Li, Chao Shen, Sicong Yang, Zhangang Han

**Affiliations:** 1 School of Systems Science, Beijing Normal University, Beijing, 100875, P. R. China; 2 Institute of Policy and Management, Chinese Academy of Sciences, Beijing, 100190, P. R. China; 3 College of Information System and Management, National University of Denfense Technology, Hunan, 410073, P. R. China; Tianjin University, China

## Abstract

A disastrous form of pedestrian behavior is a stampede occurring in an event involving a large crowd in a panic situation. To deal with such stampedes, the possibility to increase the outflow by suitably placing a pillar or some other shaped obstacles in front of the exit has been demonstrated. We present a social force based genetic algorithm to optimize the best design of architectural entities to deal with large crowds. Unlike existing literature, our simulation results indicate that appropriately placing two pillars on both sides but not in front of the door can maximize the escape efficiency. Human experiments using 80 participants correspond well with the simulations. We observed a peculiar property named tangential momentum, the escape speed and the tangential momentum are found to be negatively correlated. The idea to reduce the tangential momentum has practical implications in crowd architectural design.

## Introduction

Crowd stampedes happen not rarely in religious gatherings, music concerts, earthquakes or in other panic situations [1]–[3]. Recently in the literature, there emerge a great number of researches on the modeling of the behavior of pedestrians [4]–[7], such as queueing theoretical approaches [8], fluid-dynamic models [9]–[11], the social force model [2], [12]–[14], cellular automata [15]–[18], and multi-agent approaches [19], [20]. The social force model is a widely used approach [14], [21]–[25], that can model many observed collective behaviors, such as “stop-and-go”, “faster is slower”, “arching and clogging” [12], [26].

Architectural design can either facilitate or hamper the escape process in panic situations. To do this, some interesting obstacle placements were studied [12], [23], [25], [27]–[31], and the literature indicates that it is possible to increase the outflow by suitably placing an obstacle or some other shaped obstacles in front of the exit. Unsuitable obstacles, however, will slow down the flow rather than increase it. Although many researchers have already performed a significant amount of work on how to control the pedestrian flow and to maximize the escape speed in panic situations, there is no very simple but particularly effective approach to achieve the goal.

Helbing et al. [2] suggested that the obstacle may behave like a wave breaker to absorb the pressure in the crowd and to reduce it to a subcritical level. They observed the clogging effect in a pushy crowd with approximately 20 participants, while the placement of one obstacle increased the efficiency of escape. Zuriguel et al. [30] proposed that the physical mechanism behind the clogging reduction is a pressure decrease in the region of arch formation. Escobar et al. [25] compared different shaped obstacles for their ability to enhance the outflow efficiency, and pillar(s) were found to be the most efficient and the simplest design. Yanagisawa et al. [29] studied efficiency of escape with an obstacle on hexagonal cell space with human experiments and Shiwakoti et al. [22] performed a series of experiments with ants to enhance the panic escape of crowd through architectural design. Although these papers [2], [22], [23], [25], [29], [30] show clearly that pillar is a simple and effective shape of obstacle, none of them told us what is the best layout of the obstacles. Both Johansson [26] and Shukla [28] used a genetic algorithm to optimize the layout of the obstacles. Their works demonstrated that a genetic algorithm is an efficient tool for solving such types of problems. In [26], they claimed that the “useless channel” in the architecture design “has obviously no negative effect on the flow”. However, this design sacrifices the pedestrians in the “useless channel” to rescue the others, which we regard as unacceptable and unfair to the pedestrians in it.

People with an experience of running to catch a bus during rush hour in large and crowded cities such as Beijing may have the experience that people pushing from the middle are comparatively less successful to get on board the bus than those people pushing from the sides. This experience provides us with the heuristics required to study the property that benefits the people who push from the sides. Intuitively stated, the people pushing from the middle are “blocked” by the force coming from aside, which we call here the tangential momentum that will be defined in Simulation Results section. This is like the case when you pull out two balls from a bottle. When you pull out the balls one by one, the force is along the pulling rope and you can finish the task easily. When you pull them out simultaneously, the two balls interact with tangential force that is perpendicular to the pulling rope that gets them stuck. We make an assumption that the property that reduces the escape speed in a panic situation is the tangential momentum. If this assumption is true, to increase the escape speed is to find a better layout design of a room that can reduce the tangential momentum.

In this work, a genetic algorithm is used to provide the layout design of the obstacles (pillars) that can reduce the tangential momentum, the settings and parameters for the simulation are extracted from human experiments. The human experiments are used as a test of the simulated optimal layout to see whether the layout can speed up the escape. The model, on the other hand, is a prediction of the experiments, provided the simulated escape time corresponds well with the experiment outcomes.

## Results

**Figure 1:**
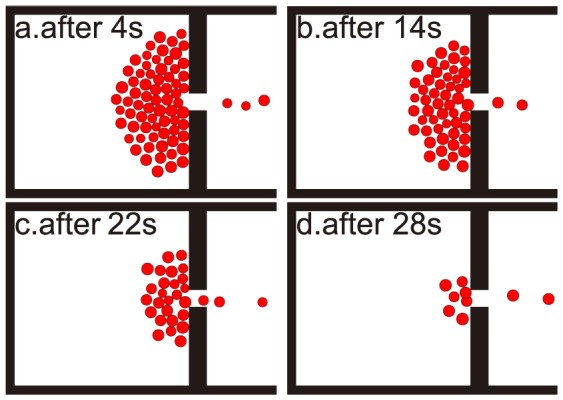
Room evacuation simulation in a panic situation with only one exit. a, b, c and d is the escape snapshot after 4, 14, 22 and 28 sec, respectively.

We consider the evacuation of a room of size 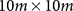 with a single exit of width of 1 m. This room might be a public hall or inside a temple, for example. We assume that there are 80 participants in this room and due to some panic situations, all the participants need to escape. We used the social force model for pedestrian dynamic. The room architecture and escape process are shown in Fig. 1, with the black line representing the wall and the red dots representing the evacuating pedestrians.

### Simulation Results

We used an Genetic Algorithm to optimize the setting of the obstacles. The simulation is run on a AMD 6172 based 2.1 GHz server. A typical optimization using 80 participants costs approximately 1,250 CPU core hours. The total computation time for our nine optimizations was 11,242 CPU core hours.

**Figure 2:**
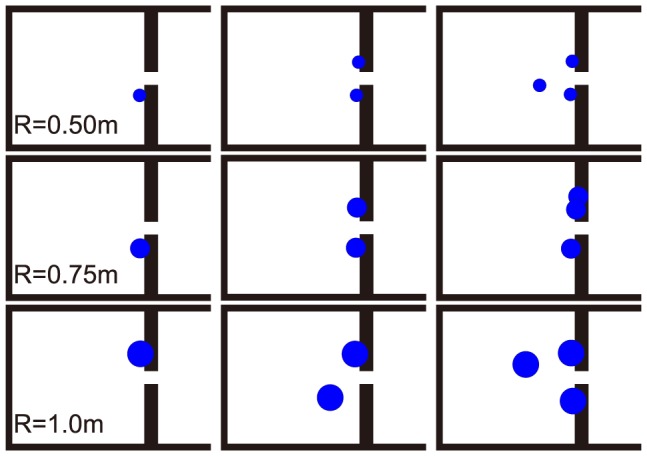
Optimized obstacle setting for 1 to 3 obstacles and 80 participants. R is the obstacle radius. The blue circle dots represent the obstacles. The GA-optimized obstacle setting results indicated that the obstacles should be near the two sides of the gate.

The optimized obstacle settings when there are 80 participants in the room are shown in Fig. 2 and Table 1 (the lower left corner of the room is set as (0,0), with the coordinates in Table 1 being in meters).

**Table 1:** GA-Optimized obstacle settings for 80 participants.

Coordinate	R		
No.	0.5 m	0.75 m	1.0 m
1	(9.63,3.72)	(9.66,3.47)	(9.69,6.80)
2	(9.91,6.23)	(9.77,6.52)	(9.64,6.78)
	(9.75,3.73)	(9.70,3.49)	(7.76,3.49)
3	(9.80,6.30)	(9.70,3.45)	(9.71,6.85)
	(9.66,3.80)	(10.25,7.39)	(9.85,3.23)
	(7.33,4.48)	(10.08,6.41)	(6.28,5.99)

The obstacle settings are the coordinate positions of the obstacle(s) in the room. No. and R represent the number and radius of the obstacles, respectively. The two numbers in each parenthesis represent the position of one obstacle, thus in the cases of 1, 2 and 3 obstacles, there are 1, 2 and 3 parentheses, respectively.

**Figure 3:**
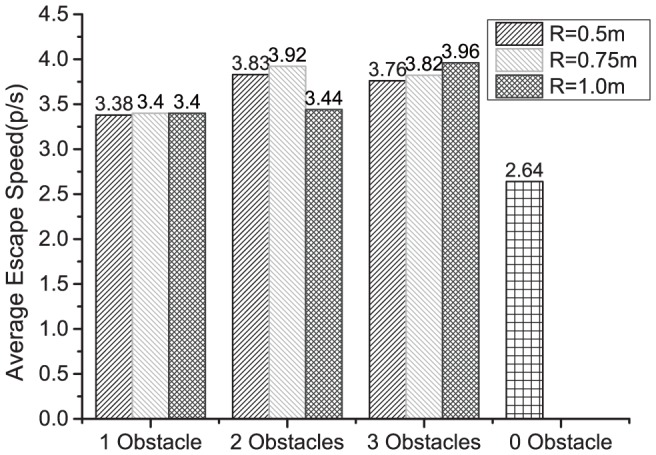
The escape speed of simulations for various obstacle configurations for 80 participants. As a baseline, when there is no obstacle in the room, the average escape speed of the crowd determined using over 100 simulations is 2.64 persons/sec, i.e., 2.64 individuals can leave the room every sec. When there are one, two or three obstacle(s) and the radius of the obstacles are 0.5 m, 0.75 m or 1 m, the average escape speed is listed on the top of the corresponding bar diagrams.

The optimization results indicate that the obstacle settings near the two sides of the gate are better than the obstacle settings studied in other literature, which are in front of the gate [2], [22], [23], [25], [29], [30]. The average escape speed is calculated as the number of pedestrians divided by the time for all pedestrians leaving the room. The escape simulation results using the above mentioned designs of obstacles in Table 1 are presented in Fig. 3. As shown in Fig. 3, the optimized obstacles can improve the pedestrians escape speed by 28% to 50%.

**Figure 4:**
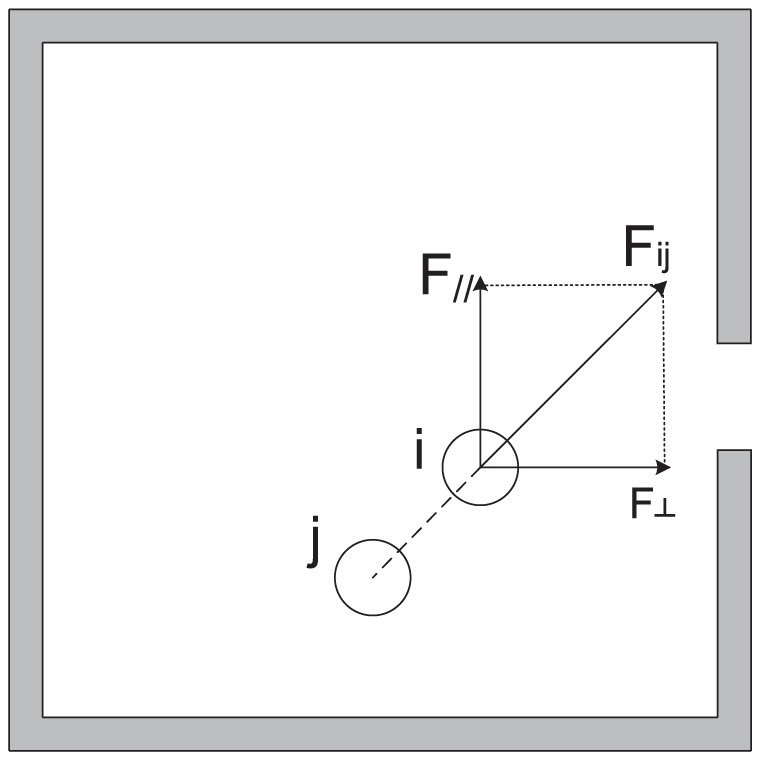
Schematic defining the tangential force. 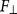
 is the component force perpendicular to the wall of the gate. 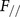 is the component force parallel to the wall. The integral of 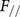 over time t is the tangential momentum. 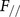 here in the figure is the same as 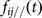 in equation (1).

We examined the “tangential momentum” in the simulations. The tangential momentum 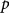 is defined by equation (1), where 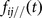 is the component force parallel to the wall of the interaction force between pedestrian 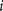 and 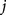 at time 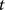, as shown in Fig. 4, and 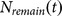 is the number of pedestrians remaining in the room at time 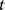.
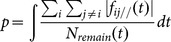
(1)


**Figure 5:**
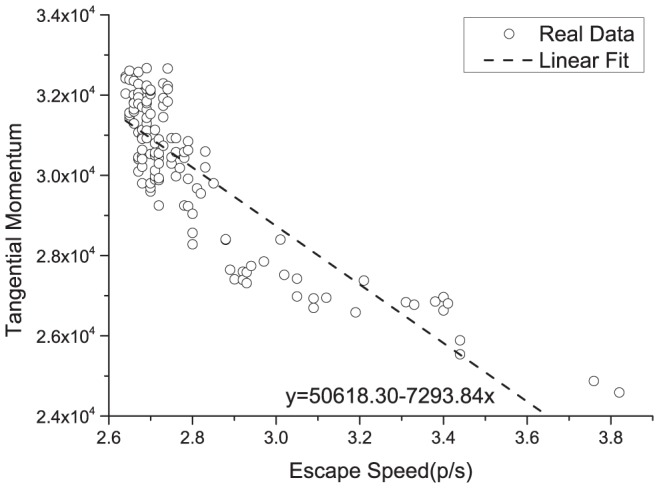
The tangential momentum vs. the escape speed for the simulation results, which comprise nine combinations of parameters of one, two or three obstacles and obstacle radius values of 0.5 m, 0.75 m or 1 m. The straight line is a linear fit to the data. We analyzed the points with an escape speed greater than 2.64 persons/sec. The reason is that the escape speed for the zero obstacle case is 2.64 persons/sec and the obstacle position configurations that reduce this escape speed are useless and not worth being analyzed. A decreasing trend for the tangential momentum, especially for high velocity values, is easily observed.

To obtain various escape speed value vs. tangential momentum samples, we need different obstacle placement configurations. During the evolutionary process of the GA, it gives plenty obstacle placement configurations which we can use. For each individual obstacle configuration, the average escape speed was calculated for over 100 escape runs. Fig. 5 is a plot of the tangential momentum vs. the escape speed for all of the simulation results, which comprise nine combinations of the parameters of one, two and three obstacles and obstacle radius values of 0.5 m, 0.75 m and 1 m. We can see a decreasing trend for the tangential momentum, especially for high velocity values.

**Figure 6:**
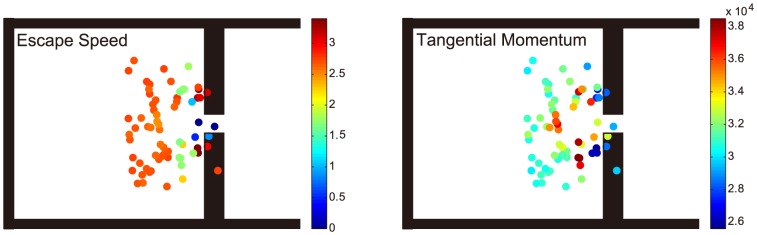
The escape speed and tangential momentum of different obstacle settings. The most efficient settings are at the side of the door but not in front of the door and the tangential momentum and the escape speed are negatively correlated.

To compare the effect of the GA-optimized obstacle setting, which is “at the side of the door”, and the “in front of the door” setting wildly used in existing literature, we used the data in the last generation of our genetic algorithm. As seen from Fig. 6, the most efficient settings with high escape speed and low tangential momentum are near the side of the door, while the settings in front of the door are less efficient. It's also shown that both the escape speed and the tangential momentum are sensitive to the position of the obstacle when the obstacle was placed quite near the door, specifically when the distance between the door and the obstacle is less than 2 m in this case, which we named “sensitive region”. However, the most efficient position of the obstacle is also in the sensitive region. If put out of the sensitive region, obstacle can not improve the escape speed by more than 5% in our simulations.

### Experimental Results

**Figure 7:**
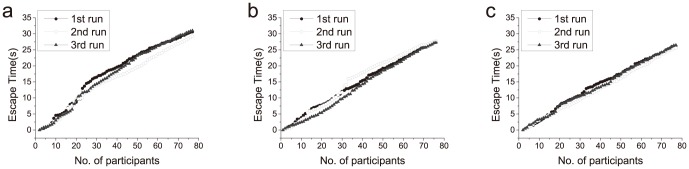
The escape time vs. the number of escaped participants for human experiments. Panels a, b and c are for zero obstacle, one obstacle and two obstacles, respectively. The data of the first run (black dots) is above the other two runs in panel a and c, which means the former participants escape slower than the latter ones. In panel b, however, the data of the first run lies between those of the second run and the third run, indicating that the escape speed of the first run is faster than that of the second run and slower than that of the third run. Therefore, the escape time has no correlation with experiments order.

To confirm our simulation results, we performed a series of human experiments. As shown in Fig. 7, the first run (black dots) in each of the three configurations is not necessarily slower (above) than the other two runs, i.e., the order of the experiments had no significant correlation with the escape speed, which indicated that there was no obvious learning effect. The experiments details was listed in Table 2. The average value for the escape speed with zero, one or two obstacles are 2.476 persons/sec, 2.732 persons/sec and 2.872 persons/sec, respectively. Using one and two obstacles increased the escape speed by 10.33% and 15.99%, respectively. The standard deviations are small, so that the use of three runs for each obstacle configuration is statistically sufficient.

**Table 2:** Average escape speed of human experiments and relevant statistical analysis.

No. of obstacles	Exp. 1	Exp. 2	Exp. 3	Mean	SD
Zero	2.448	2.561	2.419	2.476	0.075
One	2.745	2.702	2.749	2.732	0.0261
Two	2.857	2.927	2.832	2.872	0.0492

All of the human experiments used the same obstacles, which is R = 0.5 m. For each row, experiments 1, 2 and 3 are three trials of the same configuration of the obstacles. The configuration of the obstacles are the same as the simulation result. Average escape speed 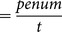, where 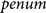 is the total participants and 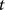 is the time for all of the participants escaping from the room.

**Figure 8:**
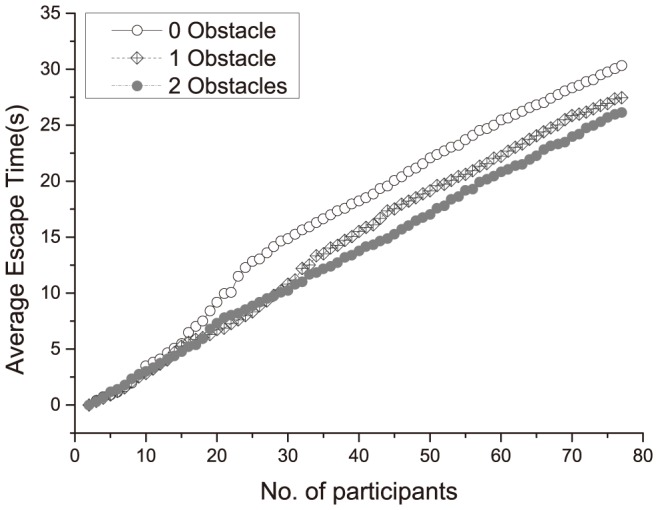
The average escape time vs. the different numbers of obstacles. The average escape time is the average time of three runs in each of the three obstacle configurations.

To clearly present the results, we show the average escape time vs. the number of escaped participants in Fig. 8. It can be seen that the difference between the obstacle numbers does not have an obvious influence on the escape speed during the initial stage of escape. When the number of escaped participants accumulated is up to approximately 15, the escape speed with obstacles is faster than that without an obstacle. When the number of escaped participants accumulated is up to approximately 35, the escape speed using two obstacles is faster than that using one obstacle. This difference could be understood if we consider the first 5 sec, when the participants near the gate run to the gate and escape before the pushing state is formed; under these conditions, the obstacles do not have an influence on the fast runners. When there is an arc formed, with the participants pushing against each other, the use of obstacle(s) helps to reduce the tangential momentum and therefore speed up the escape.

**Figure 9:**
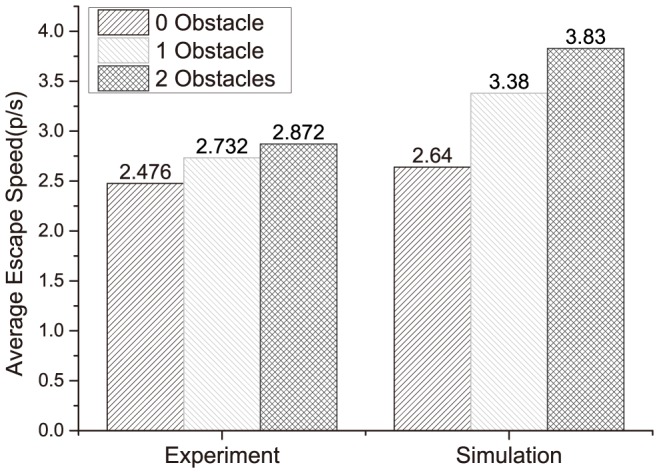
The graph showing the simulation and experimental results. The average escape speed for zero, one and two obstacles with the optimized positions is shown for the experiment (left) and the simulation (right). The chosen simulation data are the corresponding results when the obstacle radius is 0.5 m. The simulation corresponds well with the experiment in the trend that two obstacles is better than one obstacle and even better than zero obstacle.

The simulation and the experiment results are compared in Fig. 9. The simulation corresponds well with the experiment in the trend that two obstacles is better than one obstacle and still better than zero obstacle.

## Discussion

We hypothesized in this paper that the property that reduces the escape speed in a panic situation is the tangential momentum. To test this hypothesis, we simulated escapes in a panic situation using genetic algorithms to optimize the settings of the obstacles considered in the simulations, including the number, the size and the position of the obstacles. We found that obstacles at the two sides of the door is the best configuration to increase the escape speed, which is different from the configurations of existing literature. The escape speed and the tangential momentum were found to be negatively correlated, as shown in Fig. 5, thus confirming our hypothesis. The human experiment results validated the simulation results.

The previous works got various kind of settings, some of them are more or less complicated [27], [28] and others being neat and clear but not optimized [12], [23], [25], [29]. The argument of this paper is that to obtain a high escape speed, we must base on the obstacle settings that can reduce the tangential momentum. The optimized configuration with pillars at the two sides of the gate supported the concept of reducing the tangential momentum to increase the escape speed.

Fig. 2 shows the GA optimized simulation results. It is the characteristics of a GA that it may not give the optimal solution every time, but it gives a near-optimal solution fast [32]. The simulation is very time consuming, so we get the results on a 100 run average bases. The initial participants locations and the escape processes all include random factors. These will increase the fluctuation that may not be wiped out by the 100 run average. For the two obstacles with R = 1.0 m, three obstacles with R = 0.5 m and R = 1.0 m cases, the results given by GA seem to be counter intuitive in the sense that its not symmetric. To test whether GA is worth to trust, we put the pillars standing out in the room to places near the gate so that it looks symmetric. The outcome of the escape speed is worsened by 6.6%, 17.7% and 21.7% respectively. As we mentioned in the Human Experiments section, a pillar placed in the middle of the hallway could not be well accepted in both room efficiency or engineering convenience. The optimized positions are related to both the size and number of the pillars, the above mentioned example demonstrates that large radius pillar or three pillars may yield a layout that has an outstanding pillar in the middle of the hallway. Although it is efficient in a panic escape, it may cause confusion for daily use. Therefore, our suggestion for potential engineering applications is the use of two pillars placed near the two sides of the gate.

To better understand that the two pillars at the two sides of the door can reduce the tangential momentum we can refer back to the catching bus example in Introduction section. The two pillars near the door actually “block” the people pushing from the sides, and this leads to the fact that the majority people pushing from the middle get released and can pass the relatively weakened bottleneck. This may act as a guidance for further researches in the layout design to focus on reducing tangential momentum rather than trying other approaches.

## Methods

### Simulation Models

The simulations in this work are based on the social force model [12]. We use the common annotations in this field for the ease of reading. Each pedestrian 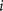 of mass 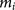 attempts to move in the direction 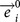 with a speed 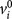, and adjusts her/his current velocity 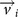 with a characteristic time 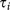. At the same time, pedestrian 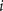 attempts to keep away from the other pedestrian 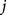, the walls 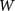, and obstacles 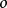, which are represented by the interaction forces: 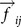, 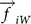, and 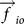, respectively [25]. Here, the obstacle social force 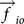 did not exist in the original social force model [12], but was introduced by Escobar [25]. 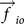 has a similar form as that of 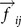 because the interaction between a pedestrian and an obstacle is very much like the interaction between pedestrians. Thus, mathematically, we have the following change in velocity by adding the last item.
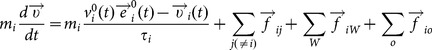
(2)



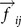
, 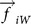 and 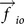 in equation (2) is explained in equations (3),(4),(5).
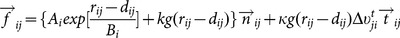
(3)

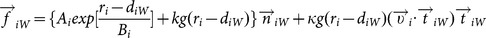
(4)

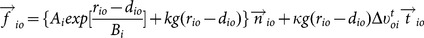
(5)


The detailed simulation parameters are available as S1 Data.

### Optimization Algorithms

Genetic algorithms have been fairly successful at solving problems of the form that are too ill-behaved (such as multimodal and/or non-differentiable) for the more conventional hill-climbing and derivative based techniques to solve. In this paper, we must optimize the position of the obstacles, which is defined in two-dimensional real number coordinates. We used a real-coded GA because it is better than binary-coded for solving this stampede problem [33].

We use the escape speed (persons/sec) as the fitness index and perform computer simulations of the panic escape process. Normally 80 people can escape from the room in less than 40 sec when there is no obstacle, if pedestrians didn′t escape from the room in 45 sec, it indicates that the obstacle setting is bad, thus there is no need to continue the escape simulation. Therefore, if the entire crowd is out of the room in less than 45 sec (it is also used by Escobar [25]), assume the time is 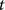, and the fitness is calculated as 
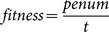
, where 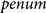 is the total participants of the crowd. If not, set 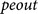 as the count of participants who are already out of the room and set 
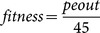
. For each obstacle setting, we take the average fitness of 100 escape simulations as the final fitness.

In addition, we propose an elitist strategy and use dynamic crossover and mutation probabilities to keep the best individual and adaptive strategies in the crossover and mutation operators to avoid early convergence[34]. The parameter settings in this current research follow [33], [34]. We set the obstacle position coordinates as the chromosome. Therefore, as an example, the chromosome of an individual in the GA population with two obstacles is 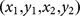. All of the chromosomes for 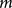 individuals with 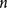 obstacles form a 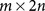 matrix representing the population. We use the Elitist strategy to retain the best individual and adaptive strategies in the crossover and mutation operators to avoid early convergence. Let 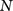, 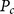, 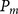, 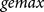 and 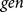 be the population size, the crossover rate, the mutation rate, the maximum generation and the current generation respectively. N is an odd integer to conveniently accommodate the Elitist strategy. The pseudo code for the GA is as follows.

1. Elitist strategy: the individual with the highest fitness value is saved and passed to the next generation automatically;
2. Select: according to the fitness value, perform a roulette wheel selection to select two individuals as parents;
3. Adaptive Crossover: set 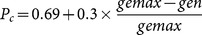, so that 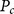 ranges from 0.69 to 0.99, which will decrease as generation increases. In this way, the algorithm can search as large a space as possible at the beginning and will not damage the good patterns in the later stage. The offspring will be generated from the crossover of individuals 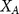 and 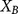 according to equation (6). Here 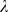 is a random number in the interval [−3, 3].

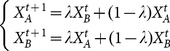
(6)If the offspring is out of the searching range, i.e., outside the room, 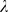 is changed to the interval [−1, 1]. Perform the crossover again until all of the offspring are in the room. Because 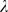 is in the interval larger than the interval [0, 1], we effectively improved the search capabilities of the genetic algorithms.

1. Adaptive Mutation: set 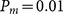. Set 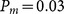 if there is no new best-individual generated for 10 consecutive generations, by doing so, we can avoid early stage convergence and improve the search capabilities of GA. To mutate one variable, for example 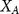, a uniform random number between [min, max] is chosen to replace 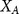.

We recalculated the 2 pillar case with tournament selection as is used in [28]. The result is the same with the roulette wheel selection as shown in S1 Fig. This means that for the current optimization problem both selection operators work well.

### Human Experiments

From the simulation we can perceive that the 2 pillar layout and the 3 pillar layout both are beneficial to the escape process in the sense that they all increase the escape speed. The best result got from the 3 pillar layout with R = 1.0 m is better than the best result of the 2 pillar layout by 1.0%. For cases with R = 0.5 m and R = 0.75 m, the 3 pillar results are not as good as the best result of the 2 pillar layout. Whats more, the real application for an optimized obstacle layout should consider not only the speed up during panic escape but also minimize the effect on the normal functions. A pillar placed in the middle of the hallway in front of the door will obviously occupy more room that could be used for accommodating more audience or for other functions. Pillars placed at the sides of the door do not have this drawback. Lastly, to place one or two pillars in front of the door has to make extra permanent engineering changes to the gym which is not accepted by any gym including the one we conducted our experiment. A removable pillar in front of the door were proved to be easily pushed away by the participants in our pre-experiments due to the huge rushing force. Hence, in the following experiments, we commit the no obstacle case, the one obstacle case and the two obstacle case.

The experiment participants were chosen among the 2012 BNU complex systems summer school. The students consisted of college undergraduate and graduate students, whose age ranging from 21 to 25 years old. The experimental site is at the second floor south exit of the BNU stadium. A 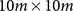 “room” is defined using rope, while leaving the gate open. Two trash barrels of size 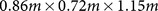 filled with water were used as the obstacles. Three cameras were used to record the experiments, with one covering the inside of the room, one covering the gate from the outside and the last one covering the gathering of the escaped participants.

#### Ethics statements

All of the subjects provided written informed consent prior to participation in the experiments and none of the subjects enrolled in our study is minor/child. The experiment protocol was approved by the Ethical Committee of SSDPP, Beijing Normal University (in accordance with the Declaration of Helsinki).

**Figure 10:**
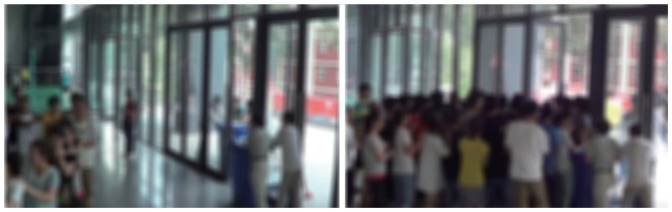
Snapshots of the experiment (blurred for privacy protection). The left panel shows the venue with the participants in the moving circling when there is an obstacle (lower right corner). The trash bin with three participants pushing outside the gate was used to ensure the gate did not collapse. The right panel shows the escape process, when the participants formed a pushing arc at the gate.

To simulate the panic condition, the participants were divided into 10 groups, with each group receiving a score according to their escape speed for each evacuation; a prize was awarded according to the ranking of the groups determined by their cumulative scores. To minimize the effect of the participants selecting their positions near the gate for each of the experimental runs, all of the participants were organized into a moving round circle, with the participants rushing to the gate when prompted by an acoustical signal. Two test runs were allotted to enable the participants to become familiar with the experiment. Subsequently, the experiment was performed for the cases of zero obstacle, one obstacle and two obstacles, with three runs for each case. Each of the 3 runs for no obstacle, one obstacle and two obstacles cases are to get rid of the fluctuations due to the initial configuration of participants location and fluctuations created in the escape process. Fig. 10 shows two of the inside screenshots. Some of the participants quit the experiment. At the end, 76 participants were recorded. S1 Video is a clip of the human experiment process. More detailed experiments data are available as S1 Data.

## Supporting Information

S1 Data All the simulation parameters and detailed experiments data are reported in the MS word file R1_SI_clean.docx.
(DOCX)

S1 Fig **The GA results of the tournament selection and the roulette wheel selection.** It shows the results of the 2 pillar case with R = 0.5 m. The coordinates for the two pillars with the tournament selection are (9.68, 6.28) and (9.92, 3.77), respectively. The coordinates for the two pillars with the roulette wheel selection are (9.91, 6.23) and (9.75, 3.73), respectively. The results are almost the same, which means that for the current optimization problem both selection operators work well.
(EPS)

S1 Video A video clip of the human experiment. It shows that the evacuation is quite close to a panic escape scenario.
(AVI)
