# Supplementary material for: Obstacle Optimization for Panic Flow - Reducing the Tangential Momentum Increases the Escape Speed
Source: PLoS One. 2014 Dec 22;9(12):e115463. doi: 10.1371/journal.pone.0115463 (PMC4274084; doi:10.1371/journal.pone.0115463)
Supplement: S1 Data — All the simulation parameters and detailed experiments data are reported in the MS word file R1_SI_clean.docx. (DOCX) [file pone.0115463.s001.docx]

# Supporting Information

## Simulation parameters

We used the parameters from [1, 2] as follows to compare the results with previous works.

$$A=2\times{10}^{3}N, B=0.08m, \tau=0.5s, k=1.2\times{10}^{5}kg\cdot s^{-2},$$

$$\kappa=2.4\times{10}^{5}kg\cdot m^{-1}\cdot s^{-1}, v_{i}^{0}=5m\cdot s^{-1}\left( i=1,2,\ldots\right), m_{i}=80kg$$

[1] Helbing D, Farkas I, Vicsek T (2000) Simulating dynamical features of escape panic. Nature 407:487–90.

[2] Escobar R, Rosa ADL (2003) Architectural design for the survival optimization of panicking fleeing victims. Adv Artif Life : 97–106.

## Experiment data

Escape time (sec) of the participants in the nine experiments. $i-j(i=0,1,2;j=1,2,3)$ denotes the $j^{th}$ experiment with $i$ obstacle(s), We set the first participant’s out time as 0.00 sec.

| No. of Participants | 0 - 1 | 0 - 2 | 0 - 3 | 1 - 1 | 1 - 2 | 1 - 3 | 2 - 1 | 2 - 2 | 2 - 3 |
| --- | --- | --- | --- | --- | --- | --- | --- | --- | --- |
| 1 | 0.00 | 0.00 | 0.00 | 0.00 | 0.00 | 0.00 | 0.00 | 0.00 | 0.00 |
| 2 | 0.40 | 0.44 | 0.40 | 0.28 | 0.20 | 0.51 | 0.34 | 0.17 | 0.46 |
| 3 | 0.56 | 0.88 | 0.76 | 0.52 | 0.48 | 0.86 | 0.74 | 0.46 | 0.80 |
| 4 | 0.80 | 1.00 | 0.96 | 1.12 | 0.56 | 1.03 | 0.97 | 0.92 | 1.60 |
| 5 | 1.12 | 1.28 | 1.08 | 1.36 | 0.84 | 1.43 | 1.26 | 1.26 | 1.66 |
| 6 | 1.68 | 1.80 | 1.44 | 1.80 | 1.32 | 1.49 | 1.54 | 1.72 | 2.12 |
| 7 | 2.28 | 1.84 | 1.72 | 2.60 | 2.00 | 1.72 | 2.06 | 2.40 | 2.57 |
| 8 | 3.72 | 2.28 | 2.16 | 3.36 | 2.16 | 2.00 | 2.35 | 2.80 | 3.15 |
| 9 | 4.56 | 2.88 | 3.00 | 3.40 | 2.84 | 2.23 | 2.75 | 3.03 | 3.32 |
| 10 | 4.64 | 3.20 | 3.72 | 3.88 | 3.32 | 2.46 | 3.15 | 3.55 | 3.37 |
| 11 | 4.80 | 3.24 | 4.24 | 4.56 | 3.76 | 2.63 | 3.49 | 3.89 | 3.78 |
| 12 | 5.16 | 4.24 | 4.64 | 5.12 | 3.80 | 3.15 | 4.06 | 4.29 | 4.00 |
| 13 | 5.52 | 4.84 | 4.88 | 5.40 | 5.40 | 3.26 | 4.69 | 4.40 | 4.06 |
| 14 | 6.04 | 5.16 | 5.20 | 6.08 | 5.96 | 3.72 | 4.86 | 4.92 | 4.58 |
| 15 | 7.00 | 6.88 | 5.52 | 6.68 | 6.00 | 4.12 | 5.66 | 4.98 | 4.98 |
| 16 | 7.96 | 7.32 | 5.88 | 7.00 | 6.36 | 4.23 | 5.72 | 5.15 | 5.21 |
| 17 | 8.32 | 7.76 | 6.44 | 7.12 | 6.44 | 4.52 | 6.64 | 5.43 | 5.72 |
| 18 | 8.36 | 8.76 | 8.12 | 7.44 | 6.76 | 4.69 | 7.26 | 6.01 | 7.09 |
| 19 | 8.64 | 9.64 | 9.24 | 7.68 | 7.24 | 5.32 | 7.61 | 6.46 | 7.84 |
| 20 | 9.64 | 9.72 | 10.48 | 7.76 | 7.44 | 5.38 | 8.35 | 6.92 | 8.12 |
| 21 | 9.72 | 9.80 | 10.76 | 8.12 | 7.76 | 5.95 | 8.58 | 7.09 | 8.35 |
| 22 | 13.00 | 10.00 | 11.52 | 8.40 | 8.08 | 6.35 | 8.81 | 7.26 | 8.47 |
| 23 | 13.72 | 11.36 | 11.84 | 8.84 | 8.48 | 6.64 | 9.21 | 7.32 | 9.09 |
| 24 | 14.56 | 12.12 | 11.88 | 9.12 | 8.80 | 7.04 | 9.55 | 7.84 | 9.21 |
| 25 | 14.84 | 12.20 | 12.20 | 9.72 | 9.40 | 7.49 | 9.67 | 8.18 | 9.72 |
| 26 | 15.28 | 12.56 | 12.96 | 9.96 | 9.96 | 7.95 | 10.01 | 8.47 | 10.07 |
| 27 | 15.92 | 13.04 | 13.52 | 10.60 | 10.28 | 8.52 | 10.35 | 8.75 | 10.12 |
| 28 | 16.28 | 13.60 | 14.04 | 10.84 | 10.96 | 8.81 | 10.64 | 9.15 | 10.35 |
| 29 | 16.52 | 13.80 | 14.36 | 11.24 | 11.60 | 9.55 | 10.70 | 9.44 | 10.52 |
| 30 | 16.96 | 14.20 | 14.68 | 12.04 | 11.76 | 9.72 | 11.50 | 10.01 | 10.87 |
| 31 | 17.04 | 14.68 | 15.16 | 12.40 | 13.92 | 10.30 | 11.95 | 10.18 | 10.93 |
| 32 | 17.48 | 14.72 | 15.56 | 12.84 | 14.12 | 10.58 | 12.93 | 10.70 | 11.44 |
| 33 | 17.76 | 15.28 | 15.96 | 12.92 | 16.00 | 11.10 | 13.21 | 10.75 | 11.61 |
| 34 | 18.32 | 15.36 | 16.20 | 13.24 | 16.08 | 11.21 | 13.44 | 11.38 | 11.67 |
| 35 | 18.64 | 15.60 | 16.76 | 13.48 | 16.32 | 12.30 | 13.50 | 11.50 | 12.24 |
| 36 | 18.80 | 16.00 | 17.24 | 13.76 | 16.64 | 12.41 | 13.96 | 11.73 | 12.53 |
| 37 | 18.96 | 16.16 | 17.64 | 14.16 | 16.92 | 13.04 | 14.36 | 12.18 | 13.04 |
| 38 | 19.24 | 16.56 | 18.04 | 14.32 | 17.20 | 13.61 | 14.53 | 12.36 | 13.21 |
| 39 | 19.60 | 16.84 | 18.28 | 15.12 | 17.68 | 13.73 | 14.93 | 12.93 | 13.38 |
| 40 | 19.88 | 16.92 | 18.80 | 15.44 | 17.88 | 14.30 | 15.10 | 13.44 | 13.90 |
| 41 | 20.12 | 17.04 | 19.32 | 15.64 | 18.12 | 14.53 | 15.39 | 13.56 | 13.96 |
| 42 | 20.80 | 17.44 | 19.76 | 16.28 | 18.60 | 15.16 | 15.67 | 13.96 | 14.30 |
| 43 | 21.08 | 17.56 | 20.12 | 17.12 | 18.96 | 15.96 | 15.90 | 14.41 | 14.36 |
| 44 | 21.68 | 17.88 | 20.68 | 17.24 | 19.24 | 16.07 | 16.07 | 14.70 | 15.04 |
| 45 | 22.20 | 18.28 | 20.72 | 17.60 | 19.64 | 16.59 | 16.24 | 14.87 | 15.96 |
| 46 | 22.28 | 18.68 | 21.64 | 17.72 | 19.88 | 17.05 | 16.70 | 15.22 | 16.24 |
| 47 | 22.84 | 18.84 | 21.80 | 18.04 | 20.24 | 17.27 | 17.27 | 15.27 | 16.82 |
| 48 | 22.96 | 19.32 | 22.36 | 18.52 | 20.52 | 17.56 | 17.39 | 15.96 | 16.87 |
| 49 | 23.36 | 19.68 | 23.20 | 18.80 | 20.64 | 17.96 | 17.45 | 16.07 | 17.50 |
| 50 | 23.80 | 19.84 | 23.52 | 19.24 | 21.00 | 18.59 | 18.13 | 16.59 | 18.08 |
| 51 | 24.32 | 20.08 | 23.76 | 19.32 | 21.20 | 18.65 | 18.53 | 16.70 | 18.19 |
| 52 | 24.40 | 20.68 | 24.04 | 19.68 | 21.36 | 19.16 | 18.93 | 17.39 | 18.82 |
| 53 | 24.56 | 20.92 | 24.16 | 20.04 | 21.76 | 19.45 | 19.05 | 17.85 | 18.99 |
| 54 | 24.92 | 21.84 | 24.36 | 20.40 | 21.80 | 19.62 | 19.68 | 18.42 | 19.39 |
| 55 | 25.52 | 22.12 | 24.64 | 20.68 | 22.20 | 19.91 | 19.73 | 18.59 | 19.62 |
| 56 | 25.72 | 22.60 | 25.24 | 20.84 | 22.64 | 20.53 | 20.65 | 19.05 | 20.08 |
| 57 | 25.84 | 22.80 | 25.48 | 21.24 | 22.76 | 20.71 | 20.82 | 19.28 | 20.36 |
| 58 | 26.16 | 23.08 | 25.76 | 21.80 | 23.20 | 21.16 | 21.05 | 19.73 | 20.59 |
| 59 | 26.40 | 23.72 | 26.24 | 21.96 | 23.40 | 21.28 | 21.39 | 19.91 | 21.16 |
| 60 | 26.56 | 23.80 | 26.52 | 22.40 | 23.72 | 21.91 | 21.68 | 20.25 | 21.22 |
| 61 | 27.04 | 24.08 | 26.84 | 22.68 | 24.08 | 22.31 | 22.14 | 20.31 | 21.74 |
| 62 | 27.12 | 24.76 | 26.92 | 22.96 | 24.12 | 22.88 | 22.19 | 20.53 | 21.79 |
| 63 | 27.40 | 24.92 | 27.44 | 23.48 | 24.68 | 23.05 | 22.31 | 20.99 | 22.54 |
| 64 | 27.60 | 25.32 | 27.56 | 23.72 | 24.88 | 23.57 | 22.59 | 21.62 | 22.59 |
| 65 | 27.64 | 25.72 | 27.76 | 24.20 | 25.24 | 23.80 | 23.11 | 22.25 | 23.05 |
| 66 | 28.00 | 25.96 | 28.28 | 24.40 | 25.56 | 24.31 | 23.45 | 22.65 | 23.39 |
| 67 | 28.36 | 26.32 | 28.48 | 24.84 | 25.80 | 24.42 | 23.57 | 22.82 | 23.57 |
| 68 | 28.40 | 26.80 | 28.96 | 25.16 | 26.28 | 25.05 | 23.62 | 22.94 | 23.91 |
| 69 | 28.80 | 26.96 | 29.28 | 25.64 | 26.48 | 25.45 | 24.14 | 23.62 | 24.20 |
| 70 | 28.96 | 27.20 | 29.60 | 25.76 | 26.52 | 25.68 | 24.31 | 23.80 | 24.42 |
| 71 | 29.52 | 27.52 | 29.68 | 26.16 | 26.60 | 25.74 | 25.00 | 24.37 | 24.88 |
| 72 | 29.60 | 27.80 | 29.84 | 26.44 | 26.84 | 26.08 | 25.23 | 24.42 | 25.23 |
| 73 | 29.96 | 28.16 | 30.32 | 26.76 | 27.08 | 26.43 | 25.57 | 24.94 | 25.45 |
| 74 | 30.20 | 28.48 | 30.56 | 26.84 | 27.24 | 26.71 | 26.03 | 25.05 | 26.03 |
| 75 | 30.48 | 28.88 | 30.84 | 27.20 | 27.68 | 27.17 | 26.14 | 25.45 | 26.37 |
| 76 | 30.64 | 29.28 | 31.00 | 27.32 | 27.76 | 27.28 | 26.25 | 25.63 | 26.48 |
